# Spring Warming Impact on the Reproductive and Vegetative Phenology and Biomass of Two Olive Cultivars in Argentina

**DOI:** 10.3390/plants15030493

**Published:** 2026-02-05

**Authors:** Leila M. Hamze, Peter S. Searles, Maria Agustina Iglesias, M. Cecilia Rousseaux

**Affiliations:** 1 Centro Regional de Investigaciones Científicas y Transferencia Tecnológica de La Rioja (CRILAR-Provincia de La Rioja-UNLaR, SEGEMAR-UNCa-CONICET), Entre Ríos y Mendoza s/n, Anillaco 5301, La Rioja, Argentina; hamze.leila@inta.gob.ar (L.M.H.); maiglesias@agro.uba.ar (M.A.I.); crousseaux@conicet.gov.ar (M.C.R.); 2 Departamento de Ciencias Exactas, Físicas y Naturales (DACEFyN), Universidad Nacional de La Rioja, Av. Luis M. de la Fuente s/n, Ciudad Universitaria de la Ciencia y de la Técnica, La Rioja 5300, La Rioja, Argentina

**Keywords:** flowering, fruit set, global warming, *Olea europaea*, pit hardening, shoot elongation

## Abstract

Olive cultivation in warm regions is likely to be vulnerable to the expected temperature increases associated with climate change. The objectives of this study were to evaluate the effects of experimental warming at the end of late winter and spring on the timing of phenological stages, the duration of developmental periods, plant growth, and biomass production. The experiment was conducted in control (T0) and warmed (+4 °C, T+) open-top chambers (OTCs) during 2018 and 2019 using two olive cultivars (‘Arbequina’, ‘Coratina’) in northwest Argentina. Warming generally led to statistically significant earlier inflorescence development, flowering, fruit set, and pit hardening, with the responses being more pronounced as the spring progressed. Earlier vegetative bud opening occurred due to warming in 2018, but not in 2019. Additionally, no differences in shoot elongation or aboveground biomass were observed due to warming at the end of spring in either 2018 or 2019. Fruit set was reduced by warming, particularly in ‘Coratina’. Overall, the experimental results show that reproductive development is more sensitive to warming than vegetative growth in young olive trees. Further studies should be conducted in a larger number of olive cultivars and regions to improve our ability to predict responses to global warming.

## 1. Introduction

Many olive-growing regions around the world are in warm and arid areas, where the crop is potentially vulnerable to expected temperature increases due to climate change [1,2,3]. Climate projections suggest that for severe consequences to be avoided in agricultural ecosystems, global temperature increases should be limited to 1.5 to 2 °C in the next several decades [4]. However, increases exceeding 4 °C are predicted in some potential scenarios [5]. Given the expected increases, it is crucial to conduct studies that provide the framework for developing adaptation strategies tailored to each productive region [6]. In this regard, the impact of increasing temperature on the timing of different vegetative and reproductive growth processes constitutes an important part of this framework.

In olive, low winter temperatures are required for flowering induction [7,8], while subsequent warm spring temperatures contribute to the forcing or thermal accumulation period that determines the full flowering date [9,10]. When peak pollen air concentrations were measured over several years in Italy, it was observed that flowering occurred earlier in years with higher spring temperatures [11]. Studies in different environments have shown that temperatures from March to May (northern hemisphere) were the best predictors of full flowering dates [12,13,14]. Therefore, olive phenological models consider thermal time accumulation as one of the main parameters for predicting the full flowering date [3,15,16].

The environmental conditions in the weeks and months prior to full flowering are important because they can influence both the duration of phenological events and flowering intensity [17]. For example, water stress during the month before full flowering reduced the number of flowers per inflorescence and fruit set, and consequently, final fruit yield decreased [18]. Furthermore, the periods from vegetative and inflorescence bud opening to full flowering in olive trees were shortened with increased temperatures in a series of field observations across seven locations in Italy over 6 years [19]. A study in western Argentina found that for every 1 °C rise in average daily temperature from late winter to early spring, the time between inflorescence bud opening and onset of flowering shortened by 4.3 days. Additionally, the period between the start and end of flowering shortened with the increase in average temperature below a threshold of 23 °C but lengthened above 23 °C [20]. In general, an increase in air temperature is associated with a shortened flowering period [21] and with a faster decline in flower fertility [22]. However, the only previous study that experimentally increased temperature found that the duration of flowering depended on the cultivar [23]. In that study, temperature was elevated throughout the entire year, including the chilling period, but the effects on inflorescence development stages were not reported. In this regard, further information during the forcing period is important to better elucidate the impact of temperature increases on inflorescence and flowering duration.

Stages after flowering can also be influenced by temperature. Pit hardening is important because it is linked to the onset of oil accumulation [24,25], and water saving strategies often use pit hardening as a developmental reference [26]. Along an altitudinal gradient, the time between the end of flowering and complete pit hardening was counterintuitively reported to be shortest at the coolest location in Calabria, Italy [27]. On the other hand, the duration between fruit set and the beginning of pit hardening was almost constant between locations, years, and cultivars in Spain [28], which suggests that the duration of this period did not depend on temperature. However, the same authors found that the length of the pit hardening period was shorter as the accumulated temperature increased. The time from fruit set to veraison (i.e., when fruits change color from green to red) was also found to be shorter in cultivar ‘Arbequina‘ when trees were warmed 4 °C above ambient temperature over the entire year, while no clear effect was observed in ‘Picual’ [23]. On the other hand, fruit growth duration was not influenced by temperature in ‘Arbequina‘ or ‘Coratina‘ when trees were warmed (3 to 4 °C) after the onset of pit hardening [29]. Taken together, the available results on phenology after flowering are not yet sufficient to make predictions as to what will occur with global warming. If earlier fruit development does occur, a shift in oil synthesis from autumn to summer could expose oil synthesis to higher temperatures that would likely negatively affect fruit oil concentration and oil quality [30,31].

It is expected that spring temperatures will influence the timing of vegetative bud opening and the vigor of shoot elongation. A study of olive trees under winter dormancy in Andalucía (Spain) found that only 1–2 weeks under warm temperatures (>20 °C) were sufficient for bud opening [32]. High temperatures also led to an increased proportion of seasonal branch elongation occurring prior to spring flowering in our previous study along a latitudinal gradient in western Argentina [20]. Similarly, vegetative biomass production was higher in trees experimentally warmed over the entire year [23] or during the summer and early fall [31]. However, these experimental warming studies did not address vegetative bud opening. A study assessing the combined effects of deficit irrigation and spring warming did report slightly more shoot elongation with warming early in the spring, but vegetative bud break and detailed aspects of reproductive phenology were not reported [33]. Thus, information on bud opening at different temperature levels in manipulative warming experiments would be important to better understand vegetative and biomass responses to temperature.

We hypothesized that warming during the end of winter and spring advances olive tree phenology by shortening the duration of reproductive developmental periods. Also, a second hypothesis was that warming during this same period leads to earlier vegetative bud opening, which extends the vegetative growth period and promotes greater biomass production. To test these two hypotheses, a warming experiment during the late winter and spring was performed in open-top chambers (OTCs) using young trees of the cultivars ‘Arbequina’ and ‘Coratina’ in two growing seasons. The experimental field station was located in arid northwestern Argentina (La Rioja; 28° S latitude, 66° W; longitude). The objectives of the experiment were to evaluate the timing of reproductive and vegetative phenological stages, duration of developmental periods, plant growth, and biomass production in both olive cultivars. The contribution of this study is principally in the simultaneous evaluation of both vegetative and reproductive phenology under experimental warming. Such information is highly relevant to understanding global warming in olive trees.

## 2. Results

### 2.1. Temperature

**Figure 1:**
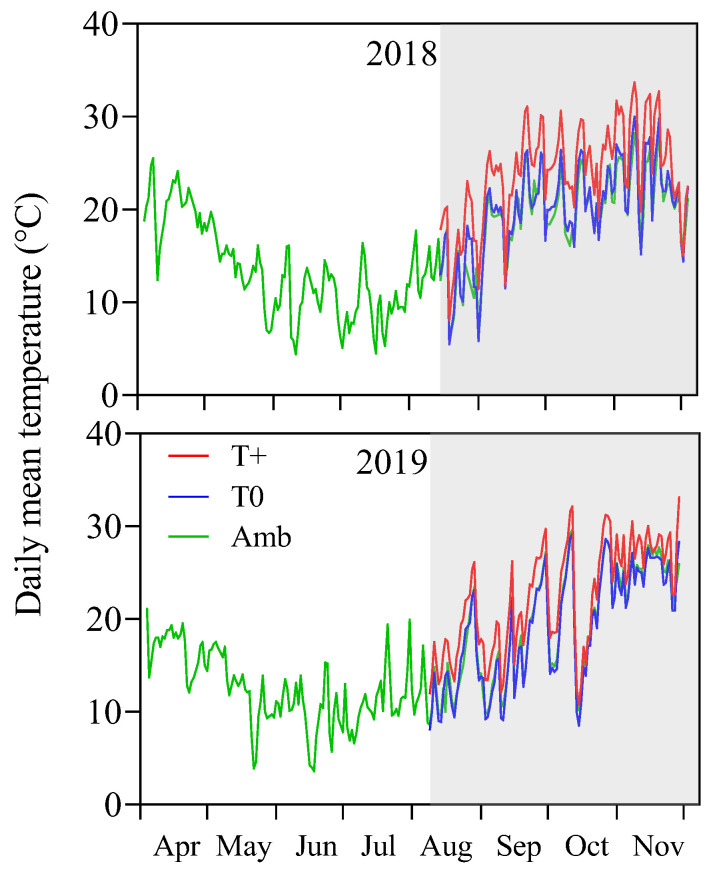
Daily mean temperature (°C) preceding and during the warming experiment in the outdoor nursery (ambient temperature, Amb) and in the warmed (T+) and control (T0) open-top chambers (OTCs). There were 8 OTCs per temperature level. The grey-shaded area indicates the warming treatment period.

The ambient temperature (Amb) averaged 13.7 °C in 2018 and 16.4 °C in 2019 in the outdoor nursery during the fall and winter months (April–July) prior to the start of the warming experiment (Figure 1). The coldest days were 11 June 2018 and 18 June 2019, when daily mean temperatures were 3.6 °C and 4.4 °C, respectively. The accumulated chilling units (CU) before the experiment were 931 CU in 2018 and 1200 CU in 2019. These values should be sufficient for normal flowering according to a commonly used olive flowering model [15]. During the warming treatment period from early August to the end of November (late winter and spring in the southern hemisphere), the daily temperature in the control OTCs (T0) averaged 20.0 °C in 2018 and 19.1 °C in 2019. These daily mean temperatures in T0 were normally no more than 1.0 °C above the Amb temperature, although maximum temperatures on sunny days in T0 were 3 to 4 °C greater than the Amb temperature values. The daily mean temperature for the warmed OTCs (T+) was 24.0 °C in 2018 and 22.2 °C in 2019, which was 4 °C and 3.2 °C above T0, respectively (*p* < 0.05). During the treatment period, temperatures (Amb, T0 and T+) were significantly higher in 2018 than in 2019 (*p* < 0.05).

### 2.2. Impact of Warming on Phenology Dates

**Figure 2:**
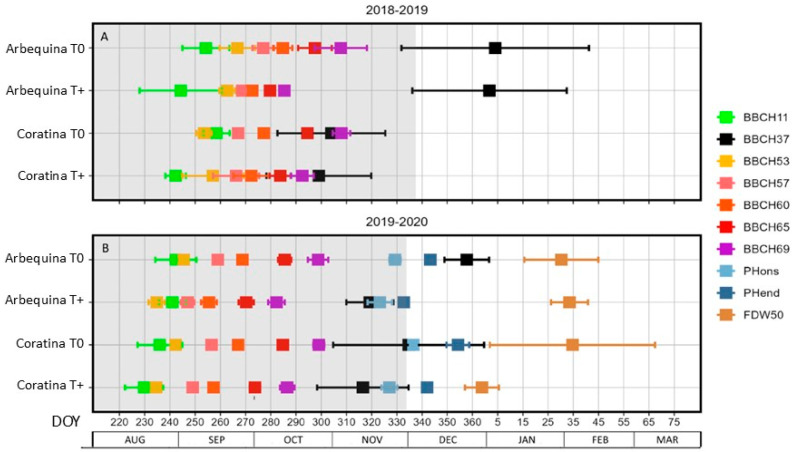
Vegetative (BBCH11 and 37) and reproductive (BBCH53, 57, 60, 65, 69, PHons, PHend, and FDW50) stage dates for two olive cultivars (‘Arbequina’; ‘Coratina’) and two temperature levels (warming treatment, T+; control, T0) during two seasons ((**A**), spring to fall of 2018–2019; (**B**), spring to fall of 2019–2020). The dates are expressed as day of the year (DOY), with 1 January of each year being DOY = 1. Phenological stages are defined according to the BBCH scale adapted for olive [34]. BBCH11: first pair of leaves completely separated, BBCH37: vegetative shoots reach 70% of their final length, BBCH53: floral bud opening, BBCH57: corolla green, BBCH60: first flowers open, BBCH65: full flowering, half of the flowers open, BBCH69: end of flowering, fruit set, PHons and PHend: onset and end of pit hardening, and FDW50: fruits with 50% of their final dry weight. Mean values ± SEs (*n* = 4) are presented. The grey-shaded area approximates the warming treatment period. Warming was from DOY 227–338 in 2018 and DOY 221–333 in 2019.

For the evaluation of the vegetative phenological stages, the date when the first pair of leaves completely separated from each other at the terminal vegetative bud (BBCH11) and the date when vegetative shoots reached 70% of their final length (BBCH37) were considered (Figure 2). BBCH11 occurred 10 and 17 days earlier in warmed trees of ‘Arbequina’ and ‘Coratina’, respectively, during spring 2018 (*p* < 0.05; Table 1). No warming effect was observed in 2019, but BBCH11 occurred 13 days earlier than in spring 2018. Similarly, there was no consistent cultivar effect between seasons, with BBCH11 in ‘Coratina’ being earlier (8.5 days) than in ‘Arbequina’ in 2019, but not in 2018. In contrast, BBCH37 was not affected by T+ in the 2018–2019 season, but it was 29 days earlier in T+ during the 2019–2020 season (*p* < 0.05). BBCH37 was much earlier (67 days) in ‘Coratina’ than ‘Arbequina’ in the 2018–2019 season. However, no difference between cultivars was apparent in 2019–2020. 

Most of the observed inflorescence and flowering developmental stages occurred earlier with warming in both cultivars and seasons (Figure 2). Only the inflorescence stages in the two-year-old trees in the spring of 2018 were not significantly earlier (Table 1). Inflorescence bud opening (BBCH53) occurred 10 and 7 days earlier in the three-year-old trees during 2019 in the warmed trees of ‘Arbequina’ and ‘Coratina’, respectively (*p* < 0.05; Table 1). Also in 2019, the stage of corolla green (BBCH57) occurred 12 and 8 days earlier in the inflorescences of the warmed trees of ‘Arbequina’ and ‘Coratina’, respectively. Both inflorescence development stages occurred earlier in ‘Coratina’ than in ‘Arbequina’ in spring 2018. Warming led to earlier flowering in the two cultivars by 8.5 (BBCH60: first flowers open), 14.5 (BBCH65: full bloom), and 18.5 (BBCH69: end of flowering) days in spring 2018, while the dates were 11, 13, and 15 days earlier with warming for the same phenological stages in spring 2019 (*p* < 0.05; Table 1). Thus, a further advancement of the phenology occurred as the season progressed. No differences between cultivars were observed for the different flowering development dates.

Fruit development could not be evaluated in the young two-year-old trees in 2018 due to little flowering intensity. When assessed in the three-year-old trees in 2019, the onset of pit hardening (PHons) occurred 6 and 9 days earlier in T+ than in T0 for ‘Arbequina’ and ‘Coratina,’ respectively (*p* < 0.05; Table 1). Furthermore, a large response was observed for the end of pit hardening (PHend), which occurred 10 days earlier in ‘Arbequina’ and 12 days earlier in ‘Coratina’ under warming (*p* < 0.05; Table 1). Cultivar also had a significant effect, with ‘Arbequina’ being on average 5 and 10 days earlier than ‘Coratina’ for PHons and PHend, respectively (Table 1). However, the FDW50 date did not show differences between treatments nor cultivars (Table 1). For this latter stage, more variability was observed compared to the other reproductive stages (Figure 2).

### 2.3. Impact of Warming on the Duration of Developmental Periods

The observed phenological stages allowed for assessing the effect of spring warming on the duration of the periods between early vegetative development (BBCH11) and three other phenological stages, including 70% of final shoot length (BBCH11-37), the beginning of inflorescence development (BBCH11-53), and full flowering (BBCH11-65). The duration of the vegetative growth period (BBCH11-37) was affected by cultivar in the 2018–19 season and by warming in the 2019–20 season (*p* < 0.05) (Table 2). The BBCH11-37 duration was 68 days shorter in ‘Coratina’ than in ‘Arbequina’ in 2018–19. On the other hand, it was 26.5 days shorter in T+ than in T0 in 2019–20. On average, the BBCH11 stage occurred 11.25 and 2 days before BBCH53 for spring 2018 and 2019, respectively, and the duration of the BBCH11-53 period was not affected by either warming or cultivar in either season (Table 2). Lastly, the duration of the BBCH11-65 period was not affected by either warming or cultivar in 2018. However, it was significantly shorter (*p* < 0.05) in T+ than in T0 for ‘Arbequina’, but not for ‘Coratina’, in 2019.

**Figure 3:**
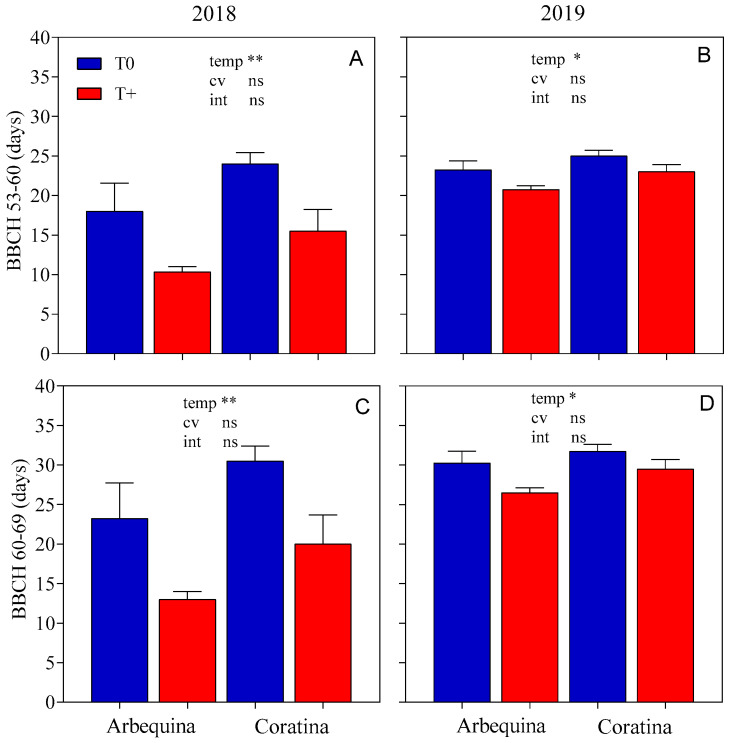
Duration (days) of inflorescence development ((**A**,**B**); BBCH 53-60) and flowering ((**C**,**D**); BBCH 60-69) for two cultivars (‘Arbequina’, ‘Coratina’) and two temperature levels (warming treatment, T+; control, T0) during two seasons (spring 2018, spring 2019). Phenological stages are BBCH53: floral bud opening, BBCH60: first flowers open, and BBCH69: end of flowering and fruit set. There were four replicate OTCs for each combination of cultivar × temperature × season. The bars represent averages ± SEs. Significant differences for temperature (temp), cultivar (cv), and their interaction (int) were evaluated for each variable using a two-way ANOVA (** *p* < 0.01, * *p* < 0.05, and ns = not significant).

In both seasons, we observed that spring warming significantly (*p* < 0.05) reduced the duration of the period between flower bud opening and the beginning of flowering (BBCH53-60) (Figure 3). This period was reduced by 8 and 2 days in T+ compared to T0 in spring 2018 and 2019, respectively. The flowering period duration varied between 13 and 31 days, with warming significantly reducing the number of days between the beginning and end of flowering (BBCH60-69) (*p* < 0.05) by 10 days in 2018 and 3 days in 2019. No differences between cultivars were observed for the duration of these periods in either season.

**Figure 4:**
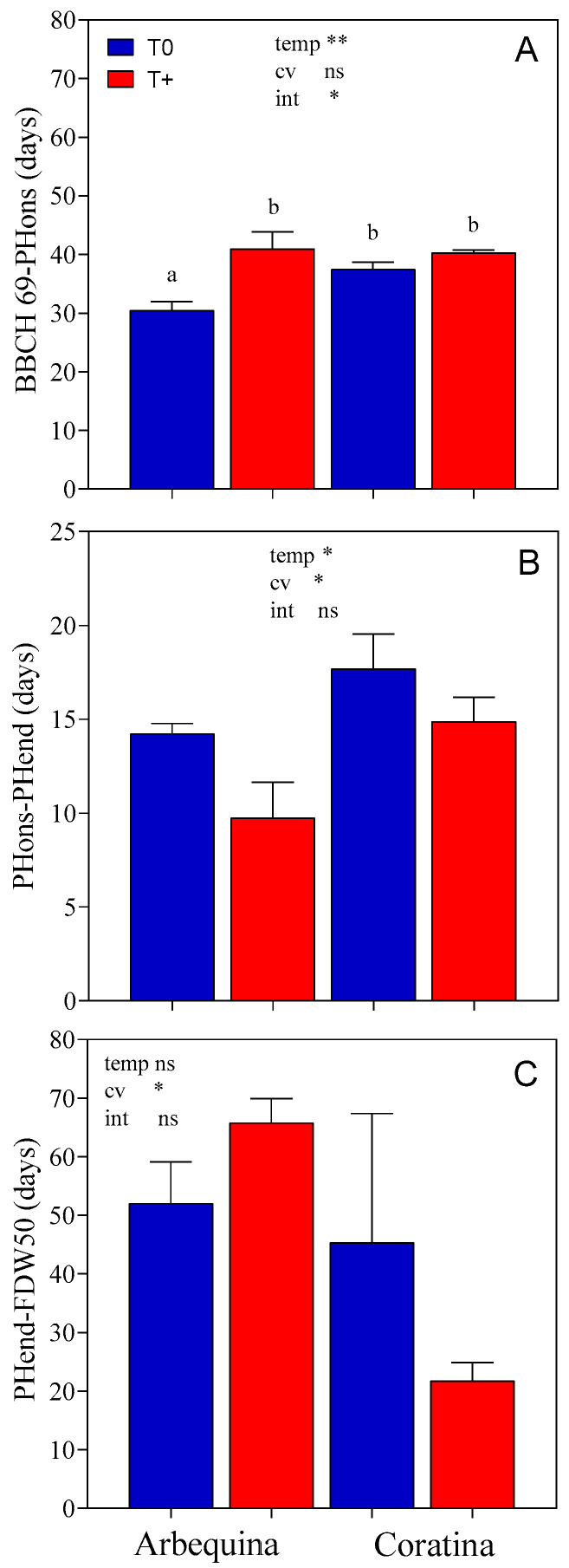
Duration (days) between the end of flowering to the onset of pit hardening ((**A**), BBCH69-PHons), the onset of pit hardening to its end ((**B**), PHons-PHend), and end of pit hardening to 50% fruit dry weight ((**C**), PHend-FDW50) for two cultivars (‘Arbequina’, ‘Coratina’) and two temperature levels (warming treatment, T+; control, T0) during the 2019–2020 season. Phenological stages are BBCH69: end of flowering and fruit set, PHons and PHend: onset and end of pit hardening, and FDW50: fruits with 50% of their final dry weight. There were four replicate OTCs for each combination of cultivar × temperature. The bars represent averages ± SEs. Significant differences for temperature (temp), cultivar (cv), and their interaction (int) were evaluated for each variable using a two-way ANOVA (** *p* < 0.01, * *p* < 0.05, and ns = not significant). Different letters indicate significant differences using LSD-Fisher (*p* < 0.05) when the temp × cv interaction was significant.

Regarding fruit development, the period between the end of flowering and the beginning of pit hardening mostly occurred during the warming treatment, while the period between the end of pit hardening and 50% fruit dry matter occurred after warming had ended. Due to the low fruit set in the two-year-old 2018 trees, these stages were only evaluated in 2019. The BBCH69-PHons period was longer in T+ (41 days) than in T0 (31 days) for ‘Arbequina’ (*p* < 0.05; Figure 4), while it lasted 39 days in ‘Coratina’ with no difference between temperature levels. On the other hand, the period from the beginning to the end of pit hardening (PHons-PHend) was significantly shortened by 3.7 days in T+ in both cultivars. An effect of cultivar was also observed with a longer period in ‘Coratina’ than ‘Arbequina’ (*p* < 0.05). The duration of PHend-FDW50 did not show significant differences between T+ and T0 for either cultivar. On average, this period lasted 59 days in ‘Arbequina’ and 33 days in ‘Coratina’ (*p* < 0.05).

### 2.4. Impact of Warming on Vegetative and Reproductive Growth

**Figure 5:**
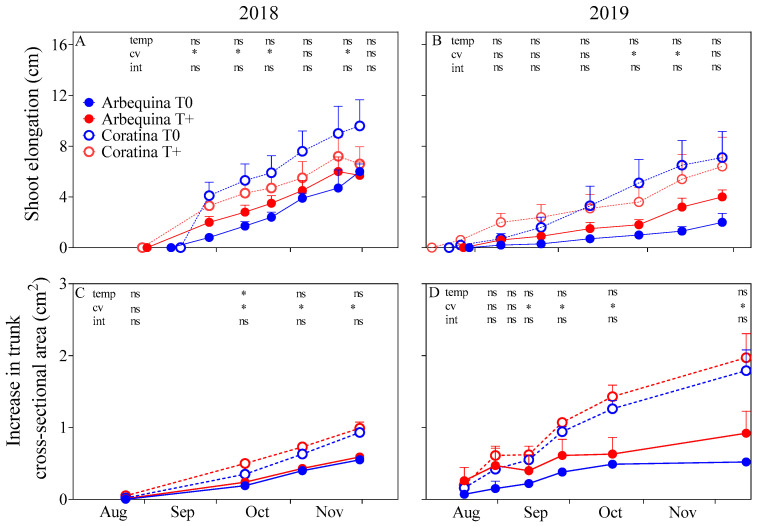
Shoot elongation (cm; (**A**,**B**)) and increase in the trunk cross-sectional area (cm^2^; (**C**,**D**)) for two cultivars (‘Arbequina’; ‘Coratina’) and two temperature levels (warming treatment, T+; control, T0) during two seasons (spring 2018, spring 2019). There were four replicate OTCs for each combination of cultivar × temperature × season. Each point represents an average ± SE. Significant differences in temperature (temp), cultivar (cv), and their interaction (int) were evaluated for each variable and measurement date using a two-way ANOVA (* *p <* 0.05 and ns = not significant).

Despite reaching BBCH11 at an earlier date in T+ in 2018, shoot elongation was similar between the T+ and T0 trees in both cultivars and seasons (Figure 5). Additionally, no warming effects were observed in the increase in trunk cross-sectional area. Both shoot elongation and the increase in trunk cross-sectional area were greater in ‘Coratina’ than in ‘Arbequina’ during most of the spring of 2018 and part of 2019 (*p* < 0.05). The increase in leaf, trunk, shoot, and total biomass that occurred during the warming period was similar between T+ and T0 in both cultivars and seasons (Table 3). A greater increase in root biomass was found in T+ with respect to T0 in 2018, but not in 2019 (*p* < 0.05). The increase in biomass of all organs was greater in ‘Coratina’ than in ‘Arbequina’ in spring 2019 (*p* < 0.05).

**Figure 6:**
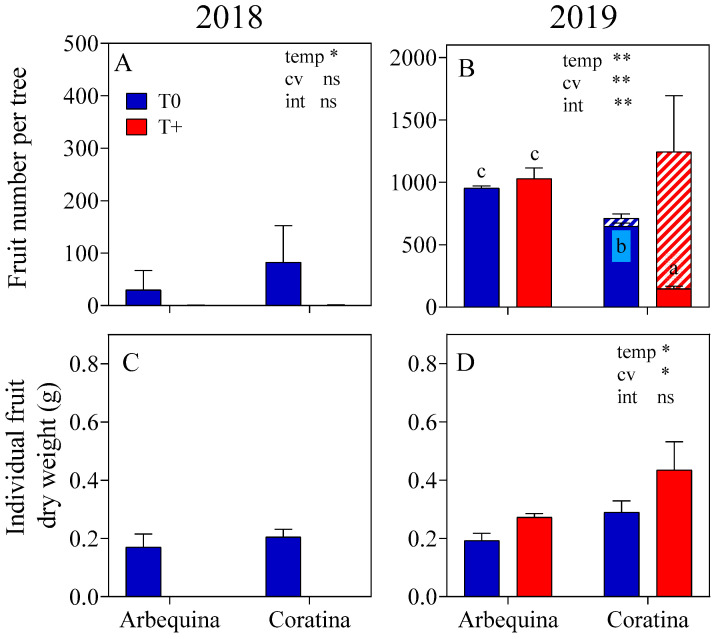
Number of normal (solid color) and parthenocarpic (striped area) fruits per tree (**A**,**B**) and individual fruit dry weight of normal fruit (**C**,**D**) for two cultivars (‘Arbequina’; ‘Coratina’) and two temperature levels (warming treatment, T+; control, T0) during two seasons (spring 2018, spring 2019). There were four replicate OTCs for each combination of cultivar × temperature × season. The bars represent averages ± SE. Significant differences in temperature (temp), cultivar (cv), and their interaction (int) were evaluated for each variable using a two-way ANOVA (** *p* < 0.01, * *p <* 0.05, and ns = not significant). Different letters indicate significant differences using LSD-Fisher (*p* < 0.05) when the temp × cv interaction was significant.

Flowering intensity was very low in the two-year-old trees in spring 2018, with less than 2.5% of the buds producing inflorescences on average in comparison to 62% in the three-year-old trees in spring 2019 (Table 4). In 2018, a significant interaction occurred with warming reducing flowering intensity in ‘Coratina’ but not in ‘Arbequina’. In 2019, no warming effects on flowering intensity were observed in either cultivar, while ‘Arbequina’ had a higher flowering intensity than ‘Coratina’. Although flowering intensity was very low, fruit set was reduced by warming in both cultivars in 2018 (Table 4). Warming negatively reduced fruit set in ‘Coratina’ by about 50% in 2019, while no effect was observed in ‘Arbequina’. When trees were harvested in December 2018, the average fruit number was 56 fruits tree^−1^ in T0. In contrast, there were no fruits present in T+ in either cultivar (Figure 6). Individual fruit dry weight was similar in the T0 fruit of both cultivars. In the 2019 harvest, ‘Coratina’ had a significantly greater number of parthenocarpic fruits in T+ than in T0 and a much lower number of normal fruits in T+ (*p* < 0.05). No difference in fruit number between T+ and T0 was observed in ‘Arbequina’. Individual fruit dry weight measured two months after full flowering was greater under T+ in both cultivars, and ‘Coratina’ fruits weighed more than those of ‘Arbequina’.

## 3. Discussion

Assessing the impact of temperature on fruit tree phenology is fundamental for understanding the potential consequences of global warming on production. In the present study, a warming treatment was applied using young, potted olive trees in open-top chambers during late winter and spring of 2018 and 2019. As proposed in our first hypothesis, experimental warming generally led to statistically significant, earlier reproductive phenological stages (BBCH53, 57, 60, 65, and 69) due to the shortening of several stages. This earlier reproductive development was found in both cultivars, ‘Arbequina’ and ‘Coratina’. These results under controlled experimental conditions help to confirm the contribution of temperature to the earlier flowering found in mature, commercial orchards at the warmer end of a latitudinal gradient (29° to 33° S) in central and northwestern Argentina, where soil type and other climatic factors varied between locations [20]. Given the similarity of the results between these two methodological approaches (i.e., experimental warming and temperature change along a latitudinal gradient), the information obtained should be relevant for modeling climate responses to global warming. With respect to the second hypothesis, although vegetative bud opening was earlier with warming in one of two seasons, vegetative biomass production was not increased, as was proposed in our second hypothesis. All of these responses are further discussed throughout this section.

Warming during late winter and spring led to reproductive stages occurring earlier for both cultivars. This included several inflorescence development (BBCH53: floral bud opening; BBCH57: corolla green) and flowering (BBCH60: first flowers open; BBCH65: full bloom, half of the flowers open; BBCH69: end of flowering and fruit set) stages. Furthermore, an accumulative effect occurred with the advances in phenology being earlier as the spring progressed. Previous observational studies in orchards in Tunisia, Argentina, and central Spain have also found earlier inflorescence development and flowering in warmer locations or years [3,20,35]. The full flowering date of experimentally warmed trees also occurred earlier than that of trees experiencing ambient temperature in both cultivars, Arbequina and Picual [23]. Similarly, full flowering occurred 19 and 31 days earlier in warmed ‘Coratina’ trees that were either well- or deficit-irrigated, respectively [33]. After flowering and fruit set, the dates of the onset and end of pit hardening continued to be earlier in the warmed trees in our experiment. In the same direction, PHons and PHend occurred 22 and 24 days earlier, respectively, in the warmest compared to the coldest location along different latitudes and altitudes in Calabria, Italy [27]. PHend is associated with the onset of mesocarp cell expansion and the beginning of the linear phase of oil accumulation [24]. In our warming experiment, the PHend stage most often occurred shortly after the end of the warming treatment, with warming leading to earlier PHend in both cultivars. This suggests an earlier start to oil synthesis due to warming.

The durations of inflorescence development (BBCH53-60) and flowering (BBCH60-69), together with the environmental conditions during both periods, are important because their durations can affect later reproductive phases [36,37]. For example, temperature effects on duration would likely affect the superposition of flowering between cultivars, which is important for ensuring cross-pollination in many olive cultivars [10]. The shortening of the BBCH53-60 and BBCH60-69 periods with warming in this study suggests that higher temperatures accelerate development both before and during flowering. Similarly, a study involving 17 olive cultivars across different locations in Italy, with mean daily temperatures in the range of 10–20 °C, observed that higher temperatures during BBCH51-61 and BBCH61-65 shortened these periods [19]. In contrast, [23] observed a lengthening in the flowering period by 8–18 days in warmed trees of ‘Arbequina’ and 7 days in ‘Picual’ in southern Spain. This may have happened because the earlier flowering dates in the warmed trees led to the flowering period in the warmed trees occurring under cooler temperatures than in the control trees. It has been observed that temperatures below 23 °C shorten flowering duration, while mean daily temperatures above 23 °C lengthen flowering for the same cultivars [20]. Thus, both shorter and longer flowering periods are possible. Lastly, given that flowering period duration is likely to vary considerably between regions [19,23], the response to warming should be examined in a greater number of regions.

Evaluating the duration of the stages before and after pit hardening is important because they are often considered when scheduling regulated deficit irrigation [26]. In our study, the duration of the period from the end of flowering to the onset of pit hardening (BBCH69—PHons) was either unaffected or even lengthened by warming. Although the PHend stage occurred 10–12 days earlier in spring 2019, this earlier date was explained by the earlier dates of the previous phenological stages rather than a shortening of the BBCH69-PHons period. Similarly, [28] in Spain and [36] in Argentina found that the duration between fruit set and the beginning of pit hardening was almost constant between locations, years, and cultivars. This suggests that temperature may not be the main factor influencing its duration [28,36]. On the other hand, the duration between PHons and PHend in our study was shortened by 3.7 days, despite not all of the pit hardening period occurring during the warming treatment. Lastly, the duration between PHend and 50% fruit size (FDW50) was not affected by temperature, as might be expected given that this period occurred after warming. As an additional observation, the duration between the end of flowering (BBCH69) and 50% fruit size showed less variation across locations in Italy due to temperature than earlier periods [19]. It is likely that modeling efforts should consider the lower temperature dependence of pit hardening and fruit growth compared to the inflorescence and flowering periods.

Our second hypothesis was that warming leads to earlier vegetative bud opening, which extends the vegetative growth period and promotes greater biomass production. Consistent with this assertion, a greater pruning biomass was reported in warmed ‘Picual’ trees [23]. However, the phenology of vegetative growth was not specifically evaluated. In our study, vegetative bud opening (BBCH11) occurred about two weeks earlier in warmed trees of both cultivars during the late winter of 2018. However, warm temperatures during the mid-winter in 2019 triggered an early bud opening soon after the start of the experiment, and no warming treatment effect on BBCH11 was observed. The BBCH37 stage, which represents the date when shoot elongation reaches 70% of its final value, was not affected by temperature in 2018, and it was actually earlier with warming in 2019. When considering the duration of BBCH11-37, there was no significant warming effect in 2018, and it was shorter by 26.5 days in 2019. Even in 2018, the earlier bud opening and slightly longer season did not lead to greater final shoot length nor trunk cross-sectional area in the warmed trees. Moreover, total vegetative biomass was similar between temperature levels. Thus, there is no evidence from these results to support the second hypothesis that warming will increase vegetative biomass produced during the spring. It is possible that the greater vegetative biomass that is sometimes observed in warm regions or under all-year-round warming treatments may be due to lower fruit loads [23] or lower fruit oil content [31]. In our study, fruit load was not decreased in ‘Arbequina’ due to warming, although it was lower in ‘Coratina’. High temperature inhibition of photosynthesis in the middle to late spring would explain the lack of a biomass increase with warming, but a limited impact of warming on photosynthesis was previously reported due to spring warming in these same cultivars [38]. The results presented here suggest that further studies related to environmental factors that control spring growth are warranted.

‘Arbequina’ is well-known as a cultivar with low vegetative vigor and is the most commonly used cultivar in super high-density hedgerow orchards, while ‘Coratina’ has greater vegetative vigor [39]. As would be expected, greater shoot elongation and an increase in trunk cross-sectional area were often observed in ‘Coratina’ compared to ‘Arbequina’ in both 2018 and 2019, and the vegetative biomass increase was greater in ‘Coratina’ in 2019. Vegetative biomass production was also greater in ‘Coratina’ than ‘Arbequina’ when evaluated during the summer and early fall under similar fruit loads [31]. In the present study, although there was no difference in the length of the vegetative growth period (BBCH11-37) between cultivars in 2019, earlier vegetative bud opening in ‘Coratina’ and a longer period between vegetative bud opening and flowering (BBCH11-65) may have contributed to greater vegetative growth in ‘Coratina’. Vegetative growth in this early period would have less competition with fruit growth than after pit hardening [40], which would favor more vegetative growth in ‘Coratina’. Genetic and architectural differences between cultivars would likely have to be considered to better evaluate growth differences and how such growth differences relate to yield production [41]. No differences in maturation time were observed between these same two cultivars across different locations in western Argentina [36].

In addition to the phenology of reproductive development, flowering intensity, fruit set, and fruit number were also evaluated. In 2018, the two-year-old trees had a very low flowering intensity, which could be due to tree age, lack of chilling units for flowering, or some combination of these factors. The accumulated chilling units (CU) up to the start of the treatments were 931 CU in 2018, which should be sufficient for normal flowering according to the flowering model of [15]. Nevertheless, the lower flowering intensity under T+ than under T0 in ‘Coratina’ would suggest that sufficient CUs had not been accumulated prior to the start of the warming treatment in 2018. It may be that very high temperatures in the fall (April, May) of 2018 substantially delayed CU accumulation more than was predicted by the model. Recent experimental evaluations of such warm spells indicate that they have the potential to drastically reduce flowering intensity [8]. When the number of chilling units was higher (1200 CU) in 2019, no response of flowering intensity to warming was observed. Additionally, the trees were one year older in 2019 (i.e., three years old), which cannot be discarded as a contributing factor to flowering intensity in young trees.

The fruit set was significantly reduced by warming in both cultivars in 2018, but only in ‘Coratina’ during 2019. In 2018, fruit set occurred during a period of high temperatures, with T+ reaching maximum temperatures of 40.6 and 42.2 °C for ‘Arbequina’ and ‘Coratina’, respectively. These high temperatures, together with the already very low flowering intensity in those two-year-old trees, resulted in almost no fruit in T+ trees at the end of the warming treatment. Previous studies have observed a decrease in pollen fertility under high temperatures above 30 °C [22,42]. In 2019, temperatures during the fruit set period (BBCH65-69) were somewhat lower, with T+ OTCs reaching maximum temperatures of 38.2 °C for ‘Arbequina’ and 37.7 °C for ‘Coratina’. In contrast to 2018, only ‘Coratina’ showed reduced fruit set under T+ in 2019. ‘Coratina’ had a large number of parthenocarpic fruits (i.e., small seedless fruit) in the T+ trees that were likely caused by reduced pollen fertility at high temperatures, which greatly reduced the number of normal fruits in these trees. In contrast, ‘Arbequina’ produced no parthenocarpic fruit under either T0 or T+ conditions. Additionally, the earlier phenology of the T+ trees in ‘Arbequina’ led to fruit set occurrence under sunny, clear sky conditions, while the fruit set of the T0 trees occurred mostly on cloudy days. Low solar radiation levels have previously been shown to reduce fruit set [43]. Thus, differences in the timing of fruit set between T+ and T0 may have resulted in the observation that ‘Arbequina’ was not affected by warming. Further research could address how fruit set is affected by warming in a larger number of cultivars.

## 4. Materials and Methods

### 4.1. Plant Material and Experimental Site

A warming experiment was conducted outdoors in open-top chambers (OTCs) during the end of winter and spring (August to December) in 2018 and 2019 using young, potted olive trees (cultivars ‘Arbequina’, ‘Coratina’) at the CRILAR-CONICET experimental station in La Rioja, Argentina (28°48′ S, 66°56′ W; 1325 m above sea level). The climate is continental with temperature variations ranging from freezing to 40 °C and an annual rainfall varying from 100 to 400 mm [20]. The trees were initially propagated from cuttings in a local commercial nursery (San Gabriel S.A., La Rioja, Argentina). They were transferred when they were one year old to the outdoor nursery at our experimental station, where they were grown in 30 L pots with a sandy soil:peat:perlite substrate (1:1:0.1,v:v:v) until being used in the experiment. During the experiment, the trees were 2 years old in 2018 and 3 years old in 2019. A new set of trees was used in 2019. They were irrigated daily and fertilized with macronutrients (15 N:15 P:15 K) monthly and with micronutrients (B, 0.02%; Cu, 0.01%; Fe, 3%; Mn, 1%; Zn, 1%; Mo, 0.007% + Mg 3% + free amino acids 5% + nitrogen 2.8%) (Aminoquelant minors, Brometan, Buenos Aires, Argentina) every two weeks. ‘Coratina’ is considered to have more vegetative vigor than ‘Arbequina’ [39], and both cultivars have low chilling requirements for flowering [44].

### 4.2. Experimental Design and Temperature Levels

The temperature treatments were applied to the potted trees in the OTCs from 15 August to 4 December 2018 (DOY 227–338) and from 9 August to 29 November 2019 (DOY 221–333). In both years, the experiment did not start until after the calculated chilling requirements had been fulfilled according to a commonly used flowering model [15]. The experimental design was a completely randomized factorial design with the two factors being cultivar and temperature in the OTCs (Figure S1). The two cultivars were ‘Arbequina’ (Arb) and ‘Coratina’ (Cor). The two temperature levels were a near-ambient temperature control (T0) and a warming treatment (T+; 4 °C above T0). There were 4 replicate OTCs of each cultivar × temperature combination (2 × 2) for a total of 16 OTCs. The combinations were: (1) ‘Arbequina’, near-ambient temperature control; (2) ‘Arbequina’, warming treatment; (3) ‘Coratina’, near-ambient temperature control; and (4) ‘Coratina’, warming treatment. Two trees of the same cultivar were placed in each OTC and used as sub-replicates. After the end of the experiment each year, one of the two trees per cultivar of each OTC was returned to the outdoor nursery to continue fruit and shoot evaluations after warming, while the other tree was harvested for biomass determinations. A new set of trees was used in 2019. A photograph of the experimental site with the OTCs is shown in Figure S2.

The OTCs used to achieve the temperature levels consisted of metal bar structures (1.5 m × 1.5 m × 2 m) covered with transparent plastic (100 µm polyethylene) on all four sidewalls. The OTCs used for the T+ treatment were equipped with two complementary heating systems, and their tops were partially covered by clear acetate (80 μm thick) to reduce the opening from 2.2 to 0.8 m^2^ to facilitate the retention of heat from the active heating systems. The first system, consisted of a 6 m long, transparent plastic sleeve that was positioned on the ground next to each T+ OTC with large, painted black stones inside. The stones increased their temperature by absorbing solar radiation during the day and the air inside the sleeve was heated as the stones re-emitted long-wave radiation. A fan pulled the hot air from the tunnel into the OTC. This system was responsible for most of the warming during the day on sunny days. A second system was necessary to maintain the desired temperature difference at night and on cloudy days. This system consisted of an electric heater connected to the OTC via a 110 mm diameter plastic tube. A control system equipped with temperature sensors (temperature controller, Cavadevices, Argentina) turned on the heaters whenever the temperature difference between a given T+ OTC and a reference T0 OTC fell below 4 °C. The T0 OTCs were equipped with an external air intake system for ventilation, but without any heating system. More details about the heating and temperature control system were described by [45]. Using both T0 and T+ OTCs allowed for different temperatures, while other environmental variables (photosynthetically active radiation, atmospheric CO_2_ concentration, wind, absolute humidity) were similar between OTC types.

In each OTC, the temperature was measured every 15 min using temperature sensors (Cavadevices, Argentina) placed 1 m above the ground at plant height. These sensors were protected by a radiation shield covered with thermal insulation and aluminum foil. The sensors were connected to a 16-channel data logger (Cavadevices, Argentina) that recorded the data for later analysis. The environmental temperature outside the OTCs (Amb) was measured similarly in the adjacent outdoor nursery. Using the Amb temperature data, the number of chilling units (CUs) accumulated for flowering was calculated prior to the start of the experiment each season to determine whether the chilling requirements had been fulfilled according to the [15] model.

### 4.3. Phenological Observations

Observations were made by a single observer every four days from August (start of treatments) until late October (end of flowering) according to the BBCH scale adapted for olive [34]. Observations were made for the vegetative stages of leaf development (BBCH07-19) and shoot development (BBCH31-37) as well as for the reproductive stages of inflorescence emergence (BBCH50-59) and flowering (BBCH60-69). To determine the phenological stages related to fruit development, successive harvests of 15 fruits per tree were conducted from late November to mid-June at intervals of 10 days in the 2019–2020 season, except for mid-January and February when no change in phenological stage was expected.

The phenological observations of the early vegetative stages (BBCH07-19) were conducted between the start of the treatments in August and the end of September, when the first leaf pairs of the season reached fully developed leaf size and shape (BBCH19). Five marked shoots per tree were used for these observations, with each shoot being visually assigned a phenological stage on each date for the two trees per OTC. The overall vegetative stage of a tree for a given date was calculated as the average of the stages of the five observed shoots. In cases in which the evaluated stage occurred between two successive observation dates, it was determined by linear interpolation [35]. Shoot elongation was determined periodically between an initial mark and the apical meristem from mid-August to the end of May the following year. These measurements were used to determine the date when each plant reached 70% shoot elongation (BBCH37). The means and standard errors of BBCH11 (first leaves fully separated and with a greenish-gray color) and BBCH37 dates were analyzed for each season, cultivar, and temperature level.

The reproductive stages of inflorescence development and flowering were observed on four randomly selected one-year-old shoots per tree, marked at the beginning of the season, for the two trees per OTC. The specific stages evaluated were: BBCH53, floral bud opening; BBCH57, expanded inflorescences with green corollas; BBCH60, first flowers open; BBCH65, full flowering at 50% of the flowers open; and BBCH69, most flowers with fallen petals, end of flowering, and initial fruit set. The overall stage of each tree on a particular date was determined as the average of the stages observed on the four shoots, and linear interpolation was used to estimate the date of occurrence when a specific stage occurred between two observation dates. Similar to the vegetative stages, the mean date of occurrence for each BBCH stage and its standard error were calculated for each combination of season, cultivar, and temperature level.

The first fruit development stage observed was pit hardening (PH). To determine PH, the force needed to cut the pit with a knife during the early fruit samplings was assessed by making a cross-sectional cut of fresh fruits using the methodology of [46]. The force was recorded for each fruit with a value ranging from 0 (cut with no resistance) to 3 (hard pit, impossible to cut manually) (Figure S3). The cutting force for a plant on a given date was determined as the average cutting force for 15 fruits per tree. Only one tree per OTC was used for fruit development, while the other tree was not sampled so that it could be used for vegetative and fruit biomass determination. The PHons date was defined as the date when the average cutting force of the sample fruits averaged 1.0, while PHend was defined as the date when it exceeded 2.5.

The second fruit development stage determined was the date when half of the final dry weight of the fruits (FDW50) was reached. After each successive harvest, the fruits were dried in an oven at 70 °C for 3 days, and the dry weight was determined. When the maximum fruit dry weight was reached for each tree, one-half of that weight was used to determine the date that corresponded to FDW50 from the linear relationship between date and dry weight for each tree. The mean dates for PHons, PHend, and FDW50 and their standard errors were calculated for each season × cultivar × temperature combination. It should be noted that according to the BBCH phenological scale, stage BBCH75 encompasses both PH and FDW50. However, it was not clear that the two stages necessarily occurred simultaneously. Therefore, for the purposes of this study, they were treated as separate variables.

### 4.4. Trunk Growth, Flowering Intensity, Fruit Set, and Biomass

The increase in trunk cross-sectional area was calculated from measurements of N-S and E-W trunk diameter at 10 cm above the soil surface in the two trees per OTC. The measurements were performed on four and six dates during the warming period in 2018 and 2019, respectively. When the trees were only two years old in 2018, the total number of inflorescences and axillary buds per tree was counted for each of the two trees per OTC, and flowering intensity was estimated as the number of inflorescences per bud. Fruits per tree were counted on 21 November 2018, towards the end of the warming period, and fruit set was estimated as the total number of fruits divided by the number of inflorescences per tree. In 2019, when the three-year-old trees were much larger, inflorescences and axillary buds were counted inside a 20 cm × 20 cm × 20 cm volume frame placed at two positions within each tree of the two trees per OTC to determine flowering intensity. Fruit set was estimated on 12 November 2019 on two one-year-old shoots per tree whose inflorescences had been previously counted (27 September 2019).

At the end of the warming treatment in both seasons, one tree per OTC was harvested, and its organs were separated into roots, trunk, shoots, leaves, and fruits. Unfertilized, parthenocarpic fruits (2–4 mm in diameter) were separated from normal fruits. Both the number of fruits and the number of leaves per tree were counted. All plant material was dried in an oven at 70 °C until a constant weight was reached, and then the dry weight was determined.

### 4.5. Statistical Analyses

Two-way factorial analysis of variance (ANOVA) was used to determine the main effects of cultivar and temperature and their interaction on the date of each phenological stage, phenological period durations, biomass production, flowering intensity, fruit number per tree, and individual fruit dry weight for each season. When the temperature × cultivar interaction was significant (*p* < 0.05), an LSD-Fisher post-test was performed. For fruit set, a similar ANOVA was used in 2019, while a Kruskal–Wallis non-parametric test was used due to lack of normality in 2018 to assess the difference between the four temperature × cultivar combinations. Shoot elongation and the increase in trunk cross-sectional area were analyzed using repeated measures ANOVA. Prior to each analysis, the assumptions of normality and homogeneity of variance were verified. The InfoStat statistical software was used (version 2020, Córdoba National University, Argentina).

## 5. Conclusions

Understanding how the phenology, growth, and biomass of olive trees respond to temperature at the end of winter and spring is fundamental for predicting the consequences of global warming for olive-growing regions. As proposed in the first hypothesis, experimental warming (4 °C) of young, potted trees in open-top chambers led to earlier inflorescence development, flowering, fruit set, and pit hardening, with the advances being more pronounced as the spring progressed. On the other hand, vegetative phenology and biomass were less sensitive to warming. Thus, our second hypothesis that warming during the end of winter and spring leads to earlier vegetative bud opening, which extends the vegetative growth period and promotes greater biomass production, was not accepted. However, ‘Coratina’ showed greater growth than ‘Arbequina’. The experimental results are consistent with phenological observations made along a latitudinal gradient (29° to 33° S) in central and northwestern Argentina [20,36]. The information obtained using these complementary methodologies indicates the importance of temperature in spring phenology and should be useful for predicting global warming responses, although information from a larger number of cultivars and regions is needed.

## Figures and Tables

**Table 1:** Date (day of the year) of vegetative and reproductive stages for two cultivars (‘Arbequina’; ‘Coratina’) and two temperature levels (warming treatment, T+; control, T0) during two seasons (spring to fall of 2018–2019; spring to fall of 2019–2020). Phenological stages are defined according to the BBCH scale adapted for olive [34]. BBCH11: first pair of leaves completely separated, BBCH37: vegetative shoots reach 70% of their final length, BBCH53: floral bud opening, BBCH57: corolla green, BBCH60: first flowers open, BBCH65: full flowering, half of the flowers open, BBCH69: end of flowering and fruit set, PHons and PHend: onset and end of pit hardening, and FDW50: fruits with 50% of their final dry weight. There were four replicate OTCs for each combination of cultivar × temperature × season. Significant differences for temperature (temp), cultivar (cv), and their interaction (int) were evaluated for each variable using a two-way ANOVA (* *p <* 0.05; ns = not significant). Different letters indicate significant differences using LSD-Fisher (*p* < 0.05) when the temp × cv interaction was significant. Values above 365 correspond to dates occurring after 31 December for the given season.

Season	Cultivar	Temperature	Vegetative Development		Inflorescence Development		Flowering Development			Fruit Development		
			BBCH11	BBCH37	BBCH53	BBCH57	BBCH60	BBCH65	BBCH69	PHons	PHend	FDW50
2018–2019	Arbequina	T0	254	369	267	277	285	298	308			
	Arbequina	T+	244	367	263	269	273	280	286			
	Coratina	T0	259	304	254	267	277	295	308			
	Coratina	T+	242	299	257	266	272	284	293			
	temp		*	ns	ns	ns	*	*	*			
	cv		ns	*	*	*	ns	ns	ns			
	int		ns	ns	ns	ns	ns	ns	ns			
2019–2020	Arbequina	T0	242	358	245	259 b	268	286	299	329	343	395
	Arbequina	T+	241	319	235	247 a	256	270	282	323	333	399
	Coratina	T0	236	335	242	257 b	267	285	299	336	354	400
	Coratina	T+	230	316	235	249 a	257	274	287	327	342	364
	temp		ns	*	*	*	*	*	*	*	*	ns
	cv		*	ns	ns	ns	ns	ns	ns	*	*	ns
	int		ns	ns	ns	*	ns	ns	ns	ns	ns	ns

**Table 2:** Duration (days) between the separation of the first pair of leaves (BBCH11) and three other phenological stages (BBCH37, BBCH53, BBCH65) for two cultivars (‘Arbequina’; ‘Coratina’) and two temperature levels (warming treatment, T+; control, T0) during two seasons (2018–2019; 2019–2020). Phenological stages are BBCH11: first pair of leaves completely separated, BBCH37: vegetative shoots reach 70% of their final length, BBCH53: floral bud opening, and BBCH65: full flowering, half of the flowers open. Negative values indicate that BBCH53 occurred before BBCH11. There were four replicate OTCs for each combination of cultivar × temperature × season. Significant differences in temperature (temp), cultivar (cv), and their interaction (int) were evaluated for each variable using a two-way ANOVA (* *p <* 0.05; ns = not significant). Different letters indicate significant differences using LSD-Fisher (*p* < 0.05) when the temp × cv interaction was significant.

Season	Cultivar	Temperature	Duration Period (Days)		
			BBCH11-37	BBCH11-53	BBCH11-65
2018–2019	Arbequina	T0	115	13	44
	Arbequina	T+	123	22	39
	Coratina	T0	45	−5	36
	Coratina	T+	57	15	42
	temp		ns	ns	ns
	cv		*	ns	ns
	int		ns	ns	ns
2019–2020	Arbequina	T0	115	3	42 b
	Arbequina	T+	78	−6	30 a
	Coratina	T0	103	7	49 b
	Coratina	T+	87	5	44 b
	temp		*	ns	*
	cv		ns	ns	*
	int		ns	ns	*

**Table 3:** Biomass increase (g) of the different vegetative organs during the warming experiment for two cultivars (‘Arbequina’; ‘Coratina’) and two temperature levels (warming treatment, T+; control, T0) during two seasons (spring 2018, spring 2019). There were four replicate OTCs for each combination of cultivar × temperature × season. Significant differences for temperature (temp), cultivar (cv), and their interaction (int) were evaluated for each variable using a two-way ANOVA (** *p* < 0.01, * *p* < 0.05, and ns = not significant).

			Biomass Increase (g)				
Season	Cultivar	Temperature	Leaves	Trunk	Shoots	Roots	Total
	Arbequina	T0	61 ± 10	15 ± 3	42 ± 7	29 ± 13	148 ± 30
2018	Arbequina	T+	68 ± 16	18 ± 4	47 ± 10	67 ± 6	200 ± 29
	Coratina	T0	56 ± 3	21 ± 3	69 ± 25	19 ± 7	165 ± 39
	Coratina	T+	65 ± 5	25 ± 2	71 ± 17	43 ± 7	204 ± 25
	temp		ns	ns	ns	**	ns
	cv		ns	ns	ns	ns	ns
	int		ns	ns	ns	ns	ns
2019	Arbequina	T0	−65 ± 9	16 ± 5	0 ± 18	76 ± 78	27 ± 35
	Arbequina	T+	−56 ± 37	14 ± 4	12 ± 23	118 ± 23	89 ± 87
	Coratina	T0	63 ± 19	59 ± 11	170 ± 31	161 ± 55	453 ± 76
	Coratina	T+	60 ± 18	59 ± 10	155 ± 35	171 ± 30	445 ± 40
	temp		ns	ns	ns	ns	ns
	cv		**	**	**	*	**
	int		ns	ns	ns	ns	ns

**Table 4:** Flowering intensity (inflorescences bud^−1^ × 100%) and fruit set (fruits inflorescence^−1^) for two cultivars (‘Arbequina’, ‘Coratina’) and two temperature levels (warming treatment, T+; control, T0) during two seasons (spring 2018, spring 2019). There were four replicate OTCs for each combination of cultivar × temperature × season. Significant differences for temperature, cultivar (cv), and their interaction (int) were evaluated for each variable using a two-way ANOVA (** *p* < 0.01, * *p* < 0.05; ns = not significant). Different letters indicate significant differences using LSD-Fisher (*p* < 0.05) when the temp × cv interaction was significant. Due to the lack of normality for fruit set in 2018, differences in temperature level and cultivar combinations were evaluated using the Kruskal–Wallis non-parametric test.

Season	Cultivar	Temperature	Flowering Intensity (Inflorescences Bud^−1^ × 100%)	Fruit Set (Fruits Inflorescence^−1^)
2018	Arbequina	T0	0.6 a	1.94 a
	Arbequina	T+	0.1 a	0.12 b
	Coratina	T0	8.3 b	0.63 a
	Coratina	T+	0.8 a	0.12 b
	temp		*	
	cv		**	
	int		*	
2019	Arbequina	T0	80.9	0.35 a
	Arbequina	T+	81.7	0.41 a
	Coratina	T0	50.2	0.59 a
	Coratina	T+	35.4	0.28 b
	temp		ns	*
	cv		**	ns
	int		ns	**

## Data Availability

Data are available by request to the authors.

## Supplementary Materials

The following supporting information can be downloaded at: https://www.mdpi.com/article/10.3390/plants15030493/s1, Figure S1: Diagram of the completely randomized factorial design for cultivar and temperature in the open-top chambers (OTCs; squares). The two cultivars were ‘Arbequina’ (Arb) and ‘Coratina’ (Cor). The two temperature levels included a near-ambient temperature control (T0; blue squares) and a warming treatment (T+; 4°C above T0; red squares). There were 4 replicate OTCs of each cultivar × temperature combination (2 × 2) for a total of 16 OTCs. The combinations were: (1) ‘Arbequina’, near-ambient temperature control; (2) ‘Arbequina’, warming treatment; (3) ‘Coratina’, near-ambient temperature control; and (4) ‘Coratina’, warming treatment. Two trees of the same cultivar were placed in each OTC and used as sub-replicates. On the right of the diagram, the outdoor plant nursey where the trees were grown before and after the warming treatments is shown; Figure S2: Photographs of several open-top chambers (OTCs, A) along with the interior of a near-ambient control OTC (T0, B) and a warming treatment OTC (T+, C). Only the two trees on the left side of each OTC were used for this experiment. The two complementary heating systems of a T+ OTC are indicated in (A) as plastic sleeve with blackened stones (black arrow) and electric heater (red arrow); Figure S3: Fruit cross-sections with different pit hardening and related cutting resistance levels. The levels according to the methodology of [46] are: 0 = no cutting resistance; 1 = low cutting resistance; 2 = high cutting resistance; 3 = the pit cannot be cut.
