# Supplementary material for: Spring Warming Impact on the Reproductive and Vegetative Phenology and Biomass of Two Olive Cultivars in Argentina
Source: Plants (Basel). 2026 Feb 5;15(3):493. doi: 10.3390/plants15030493 (PMC12899761; doi:10.3390/plants15030493)
Supplement: Supplementary file 1 [file plants-15-00493-s001.zip › plants-4043209-supplementary.pdf]

Supplementary material

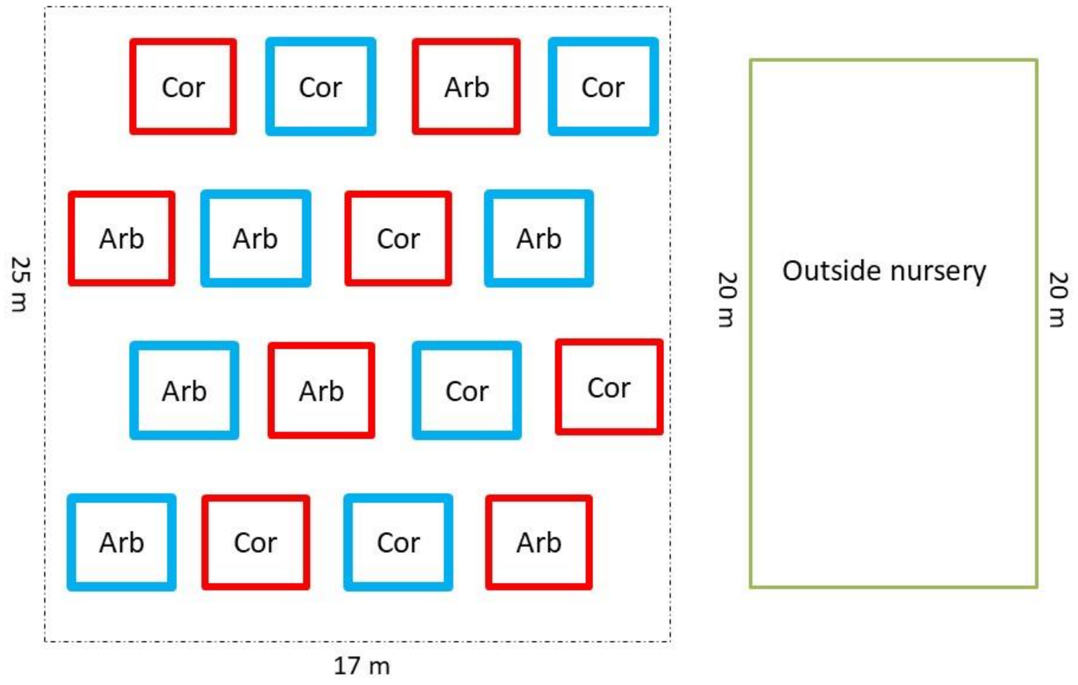

Figure S1. Diagram of the completely randomized factorial design for cultivar and temperature in the open-top chambers (OTCs; squares). The two cultivars were 'Arbequina' (Arb) and 'Coratina' (Cor). The two temperature levels included a near-ambient temperature control (T0; blue squares) and a warming treatment (T+; 4°C above T0; red squares). There were 4 replicate OTCs of each cultivar × temperature combination (2 × 2) for a total of 16 OTCs. The combinations were: 1) 'Arbequina', near-ambient temperature control; 2) 'Arbequina', warming treatment; 3) 'Coratina', near-ambient temperature control; and 4) 'Coratina', warming treatment. Two trees of the same cultivar were placed in each OTC and used as sub-replicates. On the right of the diagram, the outdoor plant nursey where the trees were grown before and after the warming treatments is shown.

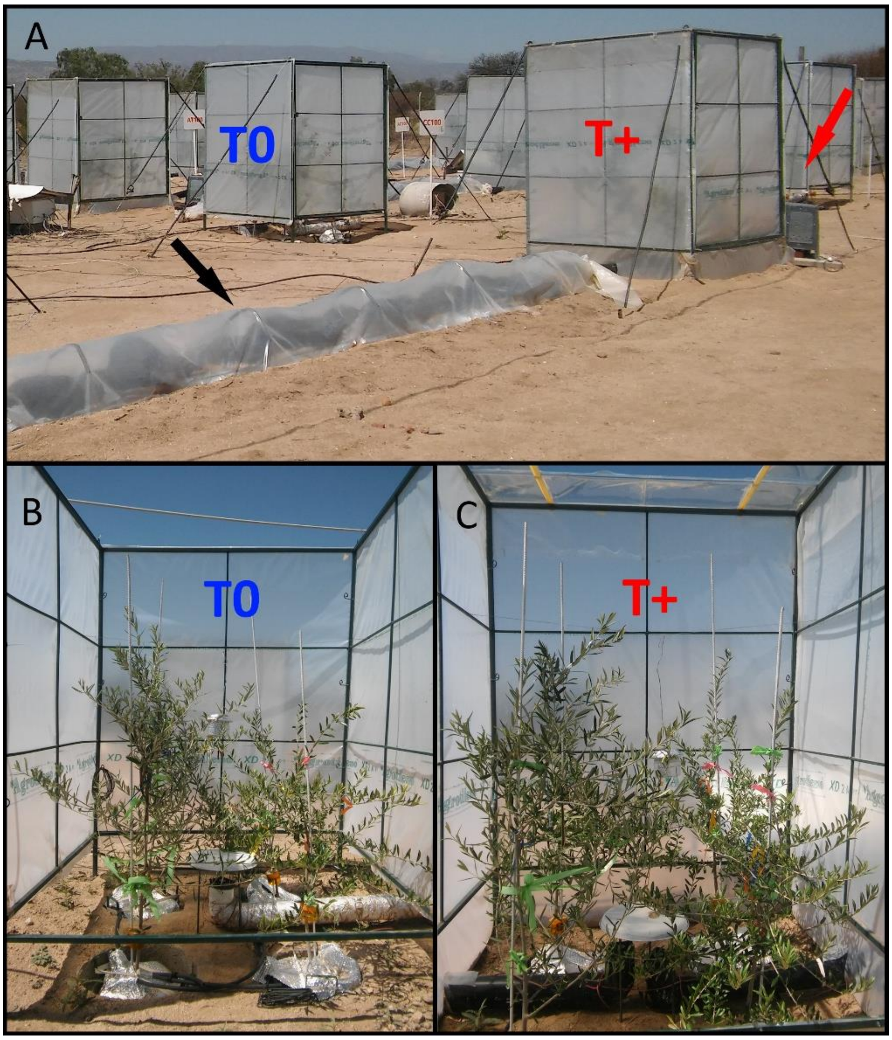

Figure S2. Photographs of several open top-chambers (OTCs, A) along with the interior of a near-ambient control OTC (T0, B) and a warming treatment OTC (T+, C). Only the two trees on the left side of each OTC were used for this experiment. The two complementary heating systems of a T+ OTC are indicated in (A) as plastic sleeve with blackened stones (black arrow) and electric heater (red arrow).

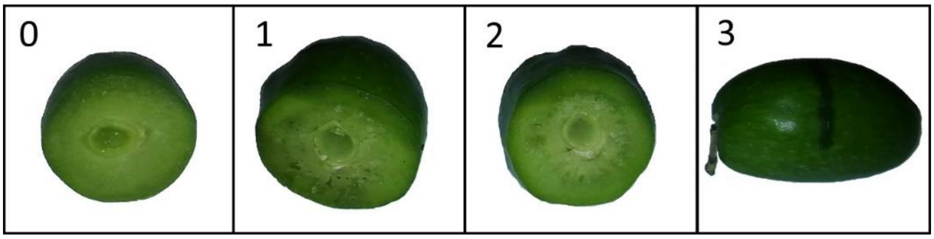

Figure S3. Fruit cross-sections with different pit hardening and related cutting resistance levels. The levels according to the methodology of [46] are: 0 = no cutting resistance; 1 = low cutting resistance; 2 = high cutting resistance; 3 = the pit cannot be cut.
